# Prognostic and functional impact of perioperative LAMA/LABA inhaled therapy in patients with lung cancer and chronic obstructive pulmonary disease

**DOI:** 10.1186/s12890-021-01537-z

**Published:** 2021-05-21

**Authors:** Yoko Azuma, Atsushi Sano, Takashi Sakai, Satoshi Koezuka, Hajime Otsuka, Naobumi Tochigi, Kazutoshi Isobe, Susumu Sakamoto, Yujiro Takai, Akira Iyoda

**Affiliations:** 1 Division of Chest Surgery, Department of Surgery, Toho University School of Medicine, 6-11-1 Omori-nishi, Ota-ku, Tokyo, 143-8541 Japan; 2 Department of Surgical Pathology, Toho University School of Medicine, 6-11-1 Omori-nishi, Ota-ku, Tokyo, 143-8541 Japan; 3 Division of Respiratory Medicine, Toho University School of Medicine, 6-11-1 Omori-nishi, Ota-ku, Tokyo, 143-8541 Japan

**Keywords:** Lung cancer, COPD, LAMA/LABA therapy, Perioperative management

## Abstract

**Background:**

Chronic obstructive pulmonary disease (COPD) is an important risk factor for postoperative complications and mortality. To determine the effects of perioperative combination therapy, using a long-acting muscarinic antagonist (LAMA) and a long-acting _2_ agonist (LABA), on preoperative lung function, postoperative morbidity and mortality, and long-term outcome in COPD patients.

**Methods:**

Between January 2005 and October 2019, 130 consecutive patients with newly diagnosed COPD underwent surgery for lung cancer. We conducted a retrospective review of their medical record to evaluate that LAMA/LABA might be an optimal regimen for patients with COPD undergoing surgery for lung cancer. All patients were received perioperative rehabilitation and divided into 3 groups according to the type of perioperative inhaled therapy and management: LAMA/LABA (n=64), LAMA (n=23) and rehabilitation only (no bronchodilator) (n=43). We conducted a retrospective review of their medical records.

**Results:**

Patients who received preoperative LAMA/LABA therapy showed significant improvement in lung function before surgery (*p*<0.001 for both forced expiratory volume in 1s (FEV_1_) and percentage of predicted forced expiratory volume in 1s (FEV_1_%pred). Compared with patients who received preoperative LAMA therapy, patients with LAMA/LABA therapy had significantly improved lung function (FEV_1_, LAMA/LABA 223.1mL vs. LAMA 130.0mL, FEV_1_%pred, LAMA/LABA 10.8% vs. LAMA 6.8%; both *p*<0.05). Postoperative complications were lower frequent in the LAMA/LABA group than in the LAMA group (*p*=0.007). In patients with moderate to severe air flow limitation (n=61), those who received LAMA/LABA therapy had significantly longer overall survival and disease-free survival compared with the LAMA (*p*=0.049, *p*=0.026) and rehabilitation-only groups (*p*=0.001, *p*<0.001). Perioperative LAMA/LABA therapy was also associated with lower recurrence rates (vs. LAMA *p*=0.006, vs. rehabilitation-only *p*=0.008).

**Conclusions:**

We believe this treatment combination is optimal for patients with lung cancer and COPD.

**Supplementary Information:**

The online version contains supplementary material available at 10.1186/s12890-021-01537-z.

## Background

Chronic obstructive pulmonary disease (COPD) is a chronic inflammatory disease of the airways characterized by persistent symptoms such as cough, sputum production, progressive breathlessness, and airflow obstruction [1]. Patients with lung cancer have a sixfold greater risk of having COPD than do matched smokers [2]. Although eligible patients with lung cancer receive a survival benefit from surgical resection, COPD is an important patient-related risk factor for postoperative complications and mortality [3]. Patients with COPD often have nonspecific airway hyperactivity, suggesting the presence of bronchospasm or latent respiratory tract infection. It is important to alleviate peripheral airway obstruction and to reduce airway secretions to improve surgical outcomes [4,6]. The combination of smoking cessation, physical therapy, and the use of bronchodilators reportedly reduces postoperative complications and improves surgical outcomes in patients with lung cancer and COPD [7, 8].

Long-acting muscarinic antagonists (LAMAs) prevent the neurotransmitter acetylcholine from binding to muscarinic receptors, leading to relaxation of the airway smooth muscle [9]. Long-acting _2_ agonists (LABAs) act on _2_-adrenergic receptors and cause relaxation of the smooth muscle [10]. Patients who receive combined LAMA/LABA therapy for COPD show superior improvement in lung function and clinical outcomes than those who receive bronchodilator monotherapy [11]. Combined LAMA/LABA therapy also leads to a lower incidence of pneumonia than the combination of inhaled corticosteroids (ICS) and a LABA [11].

Because the prognosis of patients with lung cancer complicated by COPD is reportedly poor [7], it is important to provide respiratory care for an extended duration, not just during the perioperative period. The aim of this study is to determinate the effects of perioperative LAMA/LABA therapy on preoperative lung function, postoperative morbidity and mortality, and long-term prognosis for patients with COPD and lung cancer. We hypothesize that perioperative LAMA/LABA therapy will improve surgical outcomes.

## Methods

This study was approved by the Ethics Committee of Faculty of Medicine, Toho University (A19039_27128_25095_25047).

### Study design and population

We performed a retrospective review of the medical records of patients who underwent surgical resection of lung cancer at Toho University Hospital between January 2005 and October 2019. We included patients over the age of 40 with both airflow limitation (AFL) and a smoking history of greater than 10 pack-years. Patients who had characteristics of asthma such as wheezing, shortness of breath, chest tightness, or allergic conditions were excluded. We defined AFL as a ratio of expiratory volume in 1s (FEV_1_) to forced vital capacity (FVC) less than 70%, as determined by spirometry at the patients initial visit. We excluded patients with a history of asthma or a history of inhaled therapy, and those who were missing data.

All patients with newly defined COPD received perioperative rehabilitation. The patients were divided into 3 groups according to their perioperative management: a LAMA/LABA group, a LAMA group, and a group that did not receive bronchodilators (No-BD group). The severity of AFL was classified according to the spirometric grades outlined by the Global Initiative for Chronic Obstructive Lung Disease (GOLD) [12].

Classification criteria for the three groups are based on changes in perioperative management for COPD patients as follows.

In our institute, we have executed perioperative respiratory rehabilitation and perioperative LAMA therapy for COPD patients with moderate to severe AFL since 2005 to March 2013. Since April 2013, when LABA have been developed for COPD, perioperative inhaled agent has been changed to LAMA/LABA. Eventually, therapeutic target has been expanded to COPD patients with mild AFL since July 2015. From January 2005 to June 2015, COPD patients with mild AFL, glaucoma, or severe benign prostatic hyperplasia were executed perioperative respiratory rehabilitation only.

Reassessment of respiratory function is performed on patients who received bronchodilators 1 or 2days before surgery during hospitalization. All patients use inhaled medication early in the morning and have a respiratory function test in the afternoon.

### Postoperative complications

Pneumonia was defined as the presence of at least 3 of the following: a persistent lung infiltrate on chest radiography, chest computed tomography or both; a temperature of>37.5C; and a leukocyte count>10,000/mm^3^. Acute respiratory failure was defined as postoperative ventilator dependence>12h or reintubation for mechanical ventilation. Prolonged air leakage was defined as the continuation of air leakage for more than 7days after surgery. Atrial fibrillation was diagnosed by electrocardiography and required to persist for at least 1h.

### Statistical analysis

The data were presented as the meanstandard deviation (SD) or as the median value with interquartile ranges. Categorical variables were shown as the percentage of the sample. Comparisons between 2 groups were assessed using Student's ttest for normally distributed variables or using the MannWhitney U test for nonnormally distributed variables. Differences were considered statistically significant when the *p* value was less than 0.05. Survival curves were prepared using the KaplanMeier method, and univariate comparison was performed using the log-rank test. To determine which factors were significantly associated with survival, we performed multivariate analysis using the Cox proportional hazards model. All statistical analyses were performed using JMP software, version 14.0 (SAS Institute Inc., Cary, NC, USA).

Cox hazard regression models were constructed to calculate adjusted 95% confidence intervals (CIs).

## Results

### Patient characteristics

Between January 2005 and October 2019, a total of 1330 patients with lung cancer underwent surgical resection at our institution. Of these, 192 patients (14.4%) met the inclusion criteria. A total of 62 patients had at least 1 exclusion criterion (Additional file 4: Fig.1).

A total of 130 patients with COPD who underwent surgical resection of lung cancer and who received perioperative rehabilitation were enrolled in this study. The patients were divided into 3 subgroups according to their perioperative management: the LAMA/LABA group (n=64), the LAMA group (n=23), and the No-BD group (rehabilitation only; n=43). The main patient characteristics are summarized in Table 1. Patients who received LAMA/LABA therapy were significantly older than patients in the LAMA group (*p*=0.045) and those in the No-BD group (*p*=0.027). There was no significant difference between patients in the LAMA/LABA group and the other groups with regard to sex, smoking status, pack-years of smoking or comorbidities, the type of surgical procedure performed, and histology although there was a significant difference between the LAMA/LABA group and the LAMA group on pathologic staging. Most patients underwent lobectomy (LAMA/LABA, 87.5%; LAMA, 100%; No-BD, 93.0%).

**Table 1:** Patient characteristics in all patients

	Patients with COPD (n=130)				
	LAMA/LABA	LAMA	*p* value*	No-BD	*P* value**
	n=64	n=23		n=43	
Age	73.46.7	70.66.5	0.045	70.56.2	0.027
Sex					
Male	50 (78.1)	19 (82.6)	0.644	39 (90.7)	0.088
Female	14 (21.9)	4 (17.4)		4 (9.3)	
Smoking status					
Current smoker	25 (39.0)	11 (47.8)	0.466	18 (41.9)	0.772
Ex-smoker	39 (61.0)	12 (52.2)		25 (58.1)	
Tobacco, pack-years	53.227.3	60.629.4	0.301	63.840.2	0.052
Pulmonary function					
FEV_1_ (mL)	1787.5558.8	1429.5412.5	0.005	2168.8535.2	<0.001
FEV_1_/FVC (%)	58.39.6	53. 411.4	0.049	61.410.2	0.106
FEV_1_%pred (%)	85.322.6	64.312.4	<0.001	94.62.9	0.017
Severity of AFL					
Mild	32 (50.0)	1 (4.4)	<0.001	36 (83.7)	<0.001
Moderate to severe	32 (50.0)	22 (95.6)		7 (16.3)	
Comorbidities					
Cardiovascular disease	38 (59.4)	13 (56.5)	0.812	33 (76.7)	0.575
Diabetes mellitus	17 (26.6)	6 (26.1)	0.964	12 (27.9)	0.878
Surgical procedure					
Pneumonectomy	1 (1.6)	0	0.367	0	0.844
Lobectomy	56 (87.5)	23 (100.0)		40 (93.0)	
Segmentectomy	2 (3.1)	0		1 (2.3)	
Partial resection	5 (7.8)	0		2 (4.7)	
Histology					
Adenocarcinoma	31 (48.4)	6 (26.1)	0.172	26 (60.5)	0.450
Squamous cell carcinoma	28 (43.8)	14 (60.9)		15 (34.9)	
Other	5 (7.8)	3 (13.0)		2 (4.7)	
Pathologic staging					
I	50 (78.1)	11 (47.8)	0.014	33 (76.7)	0.411
II	8 (12.5)	9 (39.1)		3 (7.0)	
III	6 (9.4)	3 (13.0)		7 (16.3)	
Recurrence (present)	7 (10.9)	11 (47.8)	<0.001	15 (34.9)	0.003

Data are presented as n (%) or as meanSD

*COPD* Chronic obstructive pulmonary disease; *LAMA* long-acting muscarinic antagonists; *LABA* long-acting 2-agonists; *BD* bronchodilator; *FEV1* forced expiratory volume in 1s; *FEV1%pred* percentage of predicted forced expiratory volume in 1s; *FVC* forced vital capacity; *AFL* airflow limitation

^*^Significance of LAMA/LABA versus LAMA

^**^Significance of LAMA/LABA versus No-BD

Pulmonary function at the initial visit was significantly worse in the LAMA group than in the LAMA/LABA group, as measured by FEV_1_, FEV_1_/FVC, and the percentage of predicted FEV_1_ (FEV1% pred) (*p*<0.05 for all). The LAMA group had a higher proportion of patients with moderate to severe AFL than the LAMA/LABA group (*p*<0.001), while the No-BD group included a higher proportion of patients with mild AFL (*p*<0.001).

### Perioperative inhaled therapy

The components of perioperative inhaled therapy are provided in Additional file 1: Table 1. In the LAMA/LABA group, patients received a LAMA plus a LABA (n=11) or a combined LAMA/LABA agent (n=53). All patients in the LAMA group received inhaled tiotropium bromide hydrate. The average duration of preoperative inhalation therapy in the LAMA/LABA group and the LAMA group was 27.7days and 24.5days (*p*=0.500), respectively. The postoperative inhalation period was 396.6days and 827.2days (*p*=0.002), respectively (Additional file 1: Table 1).

### Improvement in lung function with preoperative inhaled LAMA/LABA therapy

**Fig. 1:**
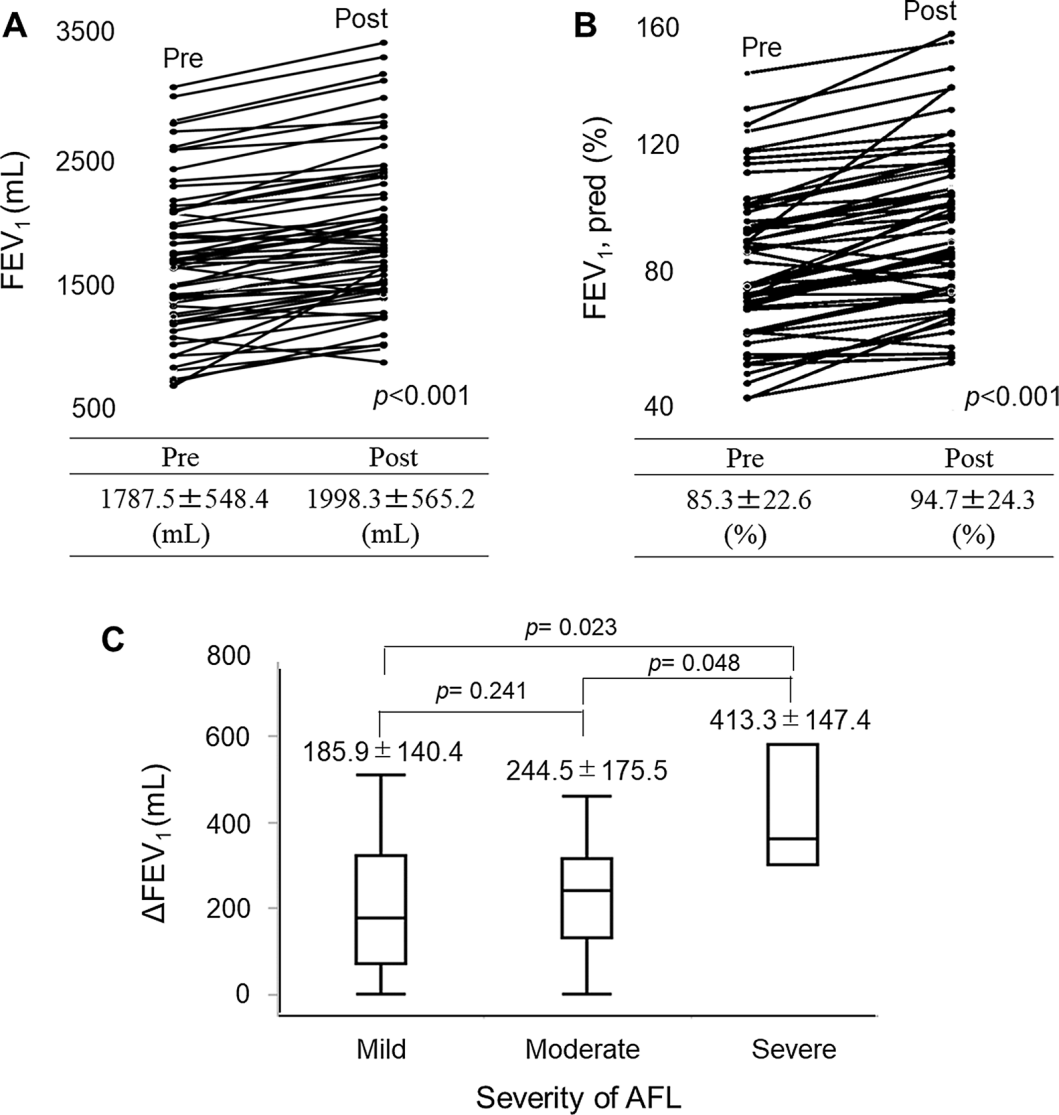
Changes in lung function with preoperative LAMA/LABA therapy. **a** Changes in forced expiratory volume in 1s (FEV_1_). **b** Changes in percentage of predicted FEV_1_ (FEV_1_%pred). **c** Improvement in FEV_1_ by severity of airflow limitation (AFL)

**Fig. 2:**
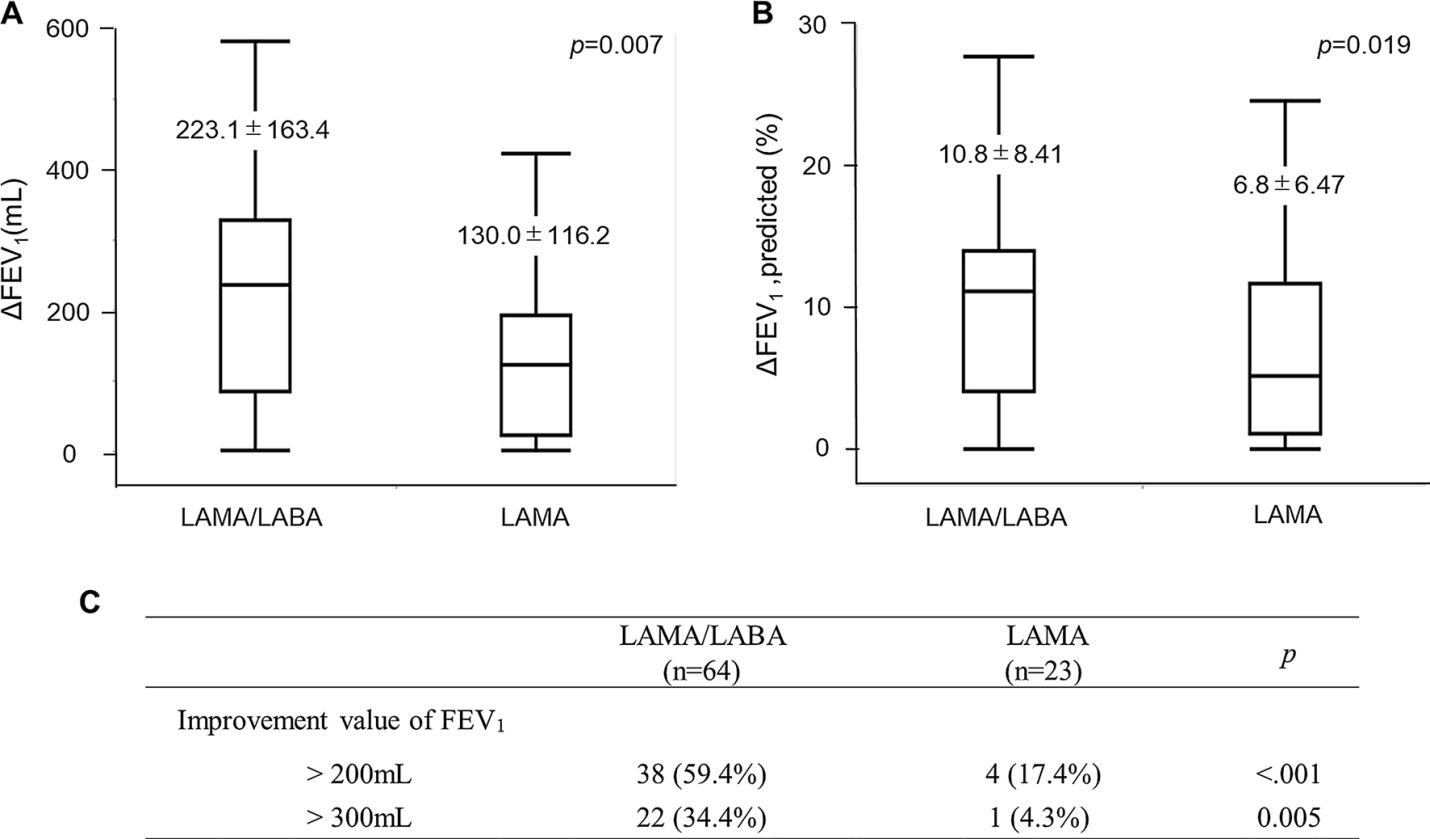
Comparison of improvement in lung function between the LAMA/LABA and LAMA groups. **a** Improvement in FEV_1_. **b** Improvement in FEV_1_%pred. **c** Proportion of patients with excellent FEV_1_ response to treatment

We reassessed lung function 1 or 2days before surgery. The values for FEV_1_ and FEV_1_%pred were significantly improved in the LAMA/LABA group (both *p*<0.001; Fig.1a, b). In the LAMA/LABA group, the treatment response tended to correlate with the severity of AFL. The patients with severe AFL showed an excellent response compared with patients with mild or moderate AFL (vs. mild AFL *p*=0.023, vs. moderate *p*=0.048; Fig.1c). We also compared the improvement in lung function between the LAMA/LABA group and the LAMA group. The increases in FEV_1_ and FEV_1_%pred were significantly higher for LAMA/LABA therapy than for LAMA therapy (FEV_1_, 223.1mL vs. 130.0mL; *p*=0.007 FEV_1_%pred, 10.8% vs. 6.8%; *p*=0.019; Fig.2a, b). The proportion of excellent treatment response was also higher in the LAMA/LABA group (FEV_1_>200mL, 59.4% vs. 17.4%; *p*<0.001 FEV_1_>300mL, 34.4% vs. 4.3%; *p*=0.005; Fig.2c).

### Postoperative morbidity and mortality

Postoperative complications and mortality are summarized in Table 2. Postoperative complications were more frequent in the LAMA group than in the LAMA/LABA group (*p*=0.007). The proportion of patients who required home oxygen therapy was higher in the LAMA group than in the LAMA/LABA group (*p*=0.008). Prolonged air leakage was more frequent in the No-BD group than in the LAMA/LABA group (*p*=0.012). Only the LAMA/LABA group had no mortality at 90days.

**Table 2:** Postoperative complications and mortality

	Patients with COPD (n=130)				
	LAMA/LABA	LAMA	*p* value*	No-BD	*p* value**
	n=64	n=23		n=43	
Complications					
Any	14 (21.9)	12 (52.2)	0.007	16 (37.2)	0.083
Pneumonia	7 (10.9)	6 (26.1)	0.081	7 (16.3)	0.422
Acute respiratory failure	1 (1.6)	2 (8.7)	0.124	2 (4.7)	0.342
Prolonged air leakage	6 (9.4)	5 (21.7)	0.126	11 (25.6)	0.012
Atrial fibrillation	4 (6.3)	2 (8.7)	0.776	4 (9.3)	0.557
Introduction of HOT	3 (4.7)	3 (13.0)	0.008	0	0.176
30-day mortality	0	0		0	
90-day mortality	0	1 (4.3)	0.093	1 (2.3)	0.246

Data are presented as n (%)

*COPD* chronic obstructive pulmonary disease; *LAMA* long-acting muscarinic antagonists; *LABA* long-acting 2-agonists; *BD* bronchodilator; *HOT* home oxygen therapy

^*^Significance of LAMA/LABA versus LAMA

^**^Significance of LAMA/LABA versus No-BD

### Survival analysis of all patients

Although a comparable proportion of patients received adjuvant chemotherapy or treatment for recurrence in all groups (Table 3), the recurrence rate was significantly lower in the LAMA/LABA group compared with both the LAMA group (*p*=0.006) and the No-BD group (*p*=0.008)(Table 3).

**Table 3:** Characteristics of patients with moderate to severe airflow limitation

	Patients with moderate to severe AFL (n=61)				
	LAMA/LABA	LAMA	*p* value*	No-BD	*p* value**
	n=32	n=22		n=7	
Age	72. 26.7	70.86.6	0.236	69.95.6	0.406
Sex			0.804		0.929
Male	27 (84.4)	18 (81.8)		6 (85.7)	
Female	5 (15.6)	4 (18.2)		1 (14.3)	
Smoking status			0.412		0.262
Current smoker	11 (34.4)	10 (45.5)		4 (57.1)	
Ex-smoker	21 (65.6)	12 (54.5)		3 (42.9)	
Severity of AFL			0.344		0.399
Moderate	29 (90.6)	18 (81.8)		7 (100)	
Severe	3 (9.4)	4 (18.2)		0	
Comorbidities					
Cardiovascular disease	16 (50.0)	13 (59.1)	0.510	6 (85.7)	0.084
Diabetes mellitus	6 (18.8)	6 (27.3)	0.459	3 (42.9)	0.170
Surgical procedure			0.098		0.887
Lobectomy	26 (81.3)	22 (100)		6 (85.7)	
Segmentectomy	1 (3.1)	0		0	
Partial resection	5 (15.6)	0		1 (14.3)	
Pathologic staging			0.016		0.148
I	26 (81.3)	11 (50.0)		4 (57.1)	
II	2 (6.3)	8 (36.4)		0	
III	4 (12,5)	3 (13.6)		3 (42.9)	
Adjuvant chemotherapy	4 (12.5)	5 (22.7)	0.322	2 (28.6)	0.286
Recurrence (present)	4 (12.5)	10 (45.5)	0.006	4 (57.1)	0.008
Treatment for recurrence					
Anticancer drug	2	7		3	
Molecular targeted drug	0	1		1	
None	2	2		0	

Data are presented as n (%) or as meanSD

*COPD* chronic obstructive pulmonary disease; *LAMA* long-acting muscarinic antagonists; *LABA* long-acting 2-agonists; *BD* bronchodilator; *AFL* airflow limitation

**Fig. 3:**
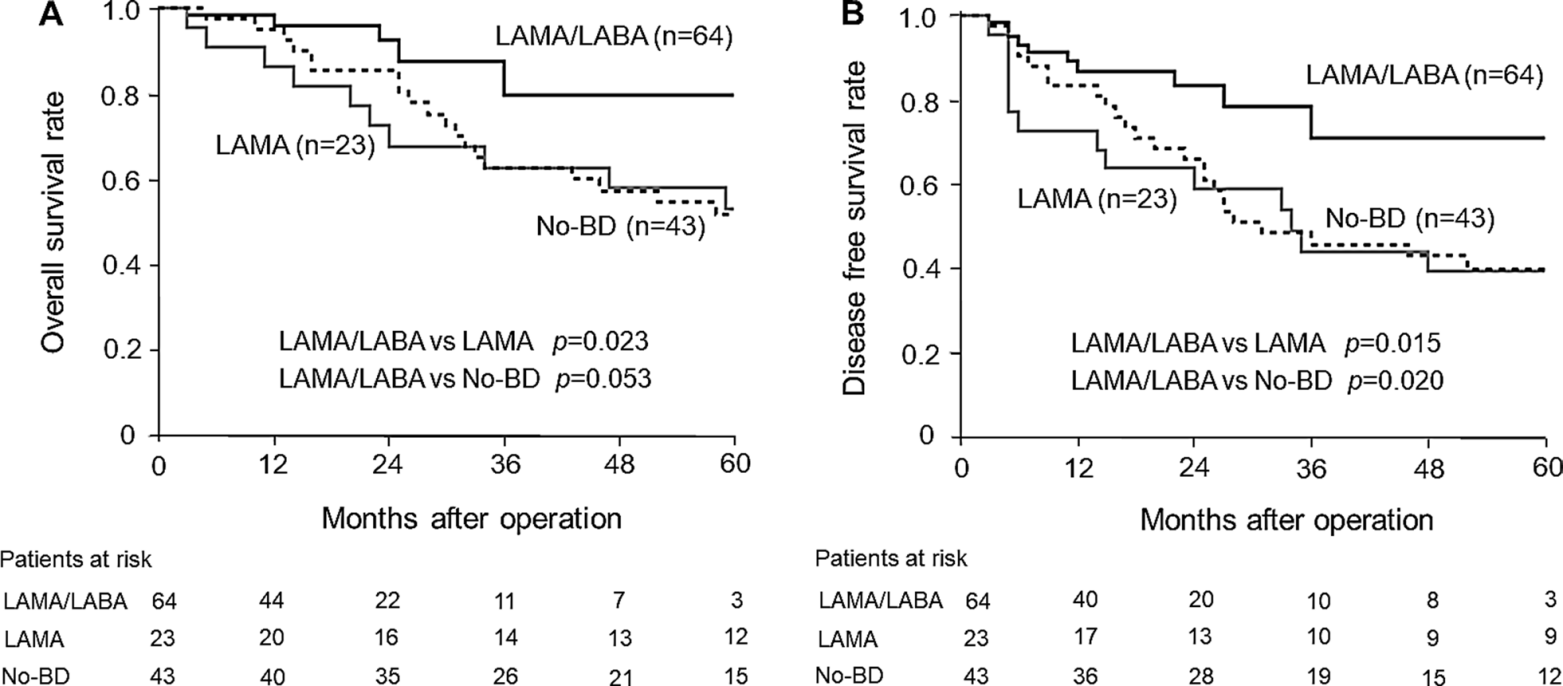
Long-term prognosis in all patients. **a** Postoperative overall survival. **b** Postoperative disease-free survival

Five patients (7.8%) in the LAMA/LABA group, 14 patients (60.8%) in the LAMA group, and 20 patients (46.5%) in the No-BD group died during the study period. The causes of death for these 39 patients are given in Additional file 2: Table 2. Lung-cancer related death was the most frequent cause in all groups. No patients in the LAMA/LABA group died of pneumonia. The cumulative OS at 5years was 79.8% in the LAMA/LABA group, 53.2% in the LAMA group, and 51.7% in the No-BD group (Fig.3a). The DFS at 5years was 70.8%, 39.2%, and 39.9%, respectively (Fig.3b). The patients in the LAMA/LABA group had significantly longer OS and DFS than patients in the LAMA group (*p*=0.023, 95%CI: 0.110.91; *p*=0.015, 95%CI: 0.160.82), and longer DFS than patients in the No-BD group (*p*=0.020, 95%CI: 0.200.89).

### Survival analysis of patients with moderate to severe AFL

We assessed the effects of perioperative inhaled LAMA/LABA therapy on the prognosis of patients with moderate to severe AFL. A total of 51 patients with moderate to severe AFL were included in the study. Of these, 32 were in the LAMA/LABA group, 12 were in the LAMA group, and 7 were in the No-BD group. Patient characteristics are given in Table 3. There was no significant difference between patients in the LAMA/LABA group and the other groups in regard to age, sex, smoking status, severity of AFL, surgical procedure, preoperative comorbidities or treatment for recurrence although there was a significant difference between the LAMA/LABA group and the LAMA group on pathologic staging. A comparable proportion of patients received adjuvant chemotherapy in all groups. As seen in the analysis of COPD patients of all severities, the recurrence rate was significantly lower in the LAMA/LABA group compared with the LAMA group (*p*=0.006) and the No-BD group (*p*=0.008; Table 3).

**Fig. 4:**
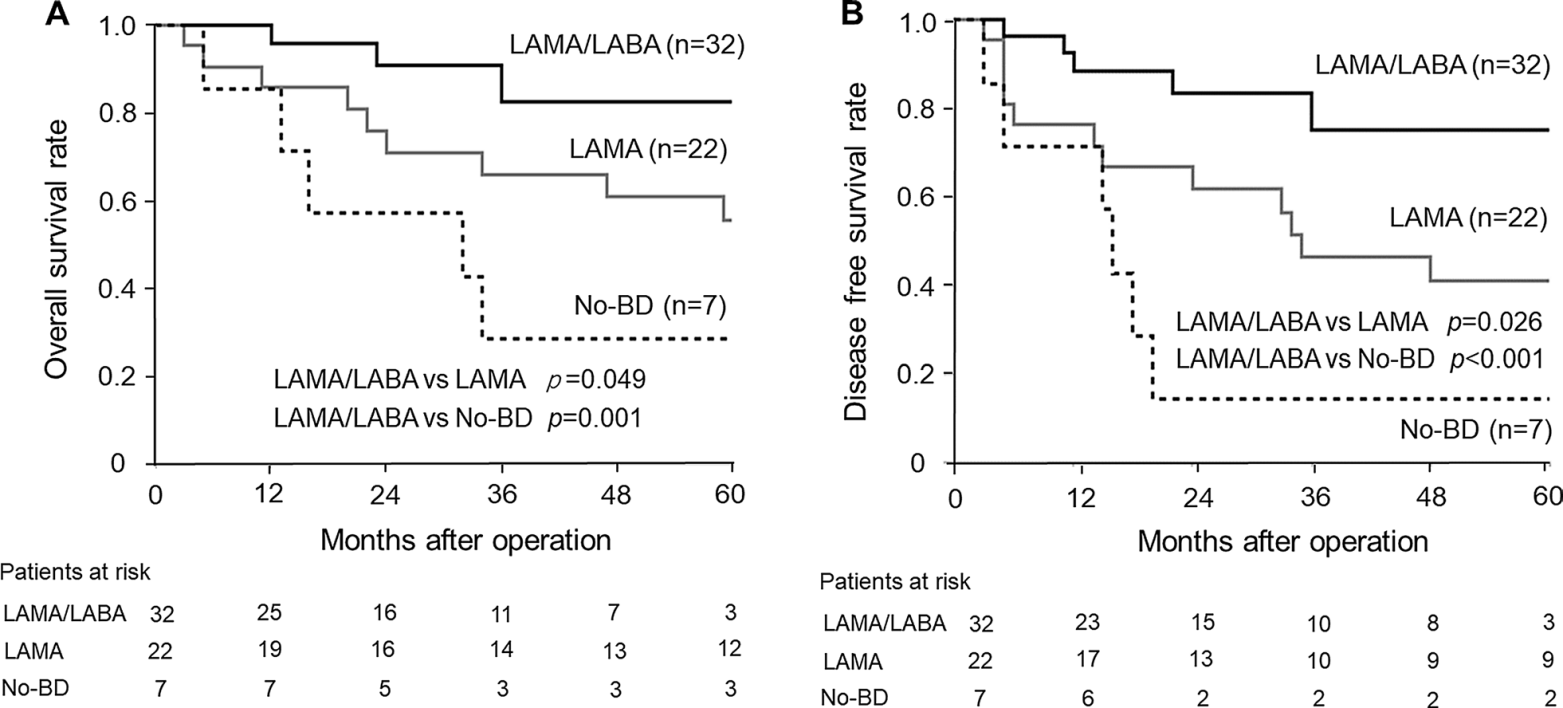
Long-term prognosis in patients with moderate to severe AFL. **a** Postoperative overall survival. **b** Postoperative disease-free survival

The OS in the LAMA/LABA, LAMA, and No-BD groups at 5years was 82.7%, 55.8%, and 28.6%, respectively (Fig.4a). The DFS at 5years was 75.3%, 41.1%, and 14.3% (Fig.4b). The patients in the LAMA/LABA group had significantly better OS and DFS compared with the LAMA group (*p*=0.049, 95%CI: 0.080.98; *p*=0.026, 95%CI:0.110.90) and with the No-BD group (*p*=0.001, 95%CI: 0.030.53; *p*<0.001, 95%CI: 0.040.45).

### Univariate and multivariate analyses of beneficial factors for long-term mortality

On multivariate analysis of all patients, histology, pathologic stage, and lymph nodes metastasis were independent prognostic factors for OS, and pathologic stage and bronchodilator were independent prognostic factors for DFS although bronchodilator was not for OS (Additional file 3: Table 3A, 3B).

On univariate analysis of patients with moderate to severe AFL, a low pathologic stage and perioperative LAMA/LABA therapy were beneficial factors for long-term mortality on univariate analysis. On multivariate analysis, perioperative LAMA/LABA therapy remained a beneficial factor (Table 4A). Only perioperative LAMA/LABA therapy was identified as a beneficial factor for recurrence on both univariate and multivariate analyses (Table 4B).

**Table 4:** Univariate and multivariate analysis of favorable factors for postoperative prognosis in patients with moderate to severe AFL

Clinicopathologic variable	Univariate analysis			Multivariate analysis		
	RR	95%CI	*p* value	RR	95%CI	*p* value
*A. Analysis of favorable factors for overall survival*						
Age (<75year)	0.88	0.292.25	0.798			
Histology:			0.083			
Squamous cell carcinoma	1.00	Reference				
Adenocarcinoma	0.47	0.181.14	0.094			
Other	0.18	0.011.01	0.051			
Lymph node metastasis (absent)	0.61	0.261.55	0.290			
Pathologic stage:			0.027			0.106
I	1.00	Reference		1.00	Reference	
II	2.96	0.998.25	0.052	2.14	0.706.16	0.176
III	3.66	1.2010.49	0.024	3.11	1.009.13	0.107
Bronchodilator			0.010			0.048
LAMA/LABA	1.00	Reference		1.00	Reference	
LAMA or No-BD	4.16	1.3711.1		3.21	1.0114.20	
*B. Analysis of favorable factors for disease-free survival*						
Age (<75year)	0.59	0.695.12	0.266			
Histology:			0.038			0.350
Squamous cell carcinoma	1.00	Reference		1.00	Reference	
Adenocarcinoma	0.42	0.170.96	0.040	0.68	0.232.05	0.311
Other	0.16	0.010.90	0.035	0.23	0.011.59	0.148
Lymph node metastasis (absent)	0.49	0.221.13	0.091			
Pathologic stage			0.007			0.233
I	1.00	Reference		1.00	Reference	
II	3.21	1.188.17	0.024	1.35	0.394.90	0.635
III	4.13	1.4711.0	0.009	2.70	0.838.37	0.278
Bronchodilator			0.003			0.018
LAMA/LABA	1.00	Reference		1.00	Reference	
LAMA or No-BD	3.90	1.5511.8		3.29	1.2210.43	0.120

*COPD* chronic obstructive pulmonary disease; *LAMA* long-acting muscarinic antagonists; *LABA* long-acting 2-agonists; *BD* bronchodilator; *RR* relative risk; *CI* confidence interval

## Discussion

In patients with lung cancer, COPD is an independent risk factor for morbidity and mortality. In the present study, we clarified the efficacy of perioperative LAMA/LABA therapy for pulmonary function, postoperative complications, and long-term prognosis in patients with newly diagnosed COPD requiring surgery for lung cancer.

The utility of perioperative bronchodilator therapy has been validated in several previous reports, but the most suitable agent has not been clarified. We previously analyzed the data of 32 patients with moderate to severe COPD and lung cancer and reported that perioperative LAMA/LABA therapy improves lung function and reduces postoperative complications to a greater degree than LAMA therapy [13]. In the present study, we assessed 130 patients with COPD of all severity levels, with similar results.

Some previous studies reported that preoperative LAMA monotherapy improves lung function or prevents postoperative respiratory complications, but other reports do not [14, 15]. Bolukbas et al. reported that adding ICS to LAMA and LABA led to improvement in lung function and fewer respiratory complications [8]. However, the use of ICS is associated with severe pneumonia [16], and steroid use is a significant risk factor for bronchopleural fistula formation [17]. Therefore, the use of perioperative ICS is controversial. Combined LAMA/LABA therapy during the perioperative period may provide a rapid and powerful bronchodilating effect, by the dual action of LAMA and LABA without adverse events such as pneumonia.

The effect of perioperative bronchodilator use on postoperative long-term prognosis in COPD patients with lung cancer has not been reported. We found that perioperative LAMA/LABA therapy is associated with a favorable prognosis compared with LAMA therapy or rehabilitation alone, especially in patients with moderate to severe COPD.

COPD is a strong promoting factor for lung cancer, and patients with COPD have poorer postoperative long-term survival due to a higher recurrence rate and poor health status [18]. Chronic inflammation of the bronchial and alveolar mucosa [19], direct effects to DNA restoration by oxidative stress [20], and genetic mutation or variation [21] are associated with COPD and the development of lung cancer. Recent reports propose that bronchodilators are able to inhibit inflammation and oxidative stress in mouse model [22]. Muscarinic receptors expressed on lung cancer cells can reportedly stimulate tumor growth via acetylcholine [23]. The M_3_ muscarinic receptor subtype is associated with cell proliferation [23], and LAMAs have the potential to inhibit the growth of lung cancer cells as M3 receptor antagonists. Li et al. reported that indacaterol induces apoptosis in lung cancer cells harboring the epidermal growth factor receptor *T790M* mutation and may be a novel candidate for treatment of gefitinib-resistant lung cancer [24]. Our results and these previous in vitro studies suggest the possibility of the dual anticancer effects of LAMA/LABAprevention of cancer development and inhibition of proliferation signals of lung cancermay contribute to a favorable prognosis and inhibition of recurrence. On the other hand, high proportion of stage I in patients who received LAMA/LABA therapy may also affect lung cancer prognosis. Randomized controlled trials would be necessary to prove the anticancer effect by LAMA/LABA therapy.

This retrospective study has limitations and biases. The duration of bronchodilator use and the individual bronchodilators employed was inconsistent. This discrepancy about selection of bronchodilators was due to the different study periods. Differences in the study period or severely of AFL between LAMA/LABA and the other groups may have affected the postoperative complications and survival rate. Finally, changes in surgical technique, surgical methods, or anesthetic agents over the years of the study period may have affected the incidence of postoperative complications.

## Conclusions

Our data demonstrate that LAMA/LABA therapy improves not only short-term outcomes such as respiratory function and postoperative complications, but also long-term prognosis in patients with lung cancer and COPD. Perioperative combined LAMA/LABA therapy is the optimal bronchodilator for patients with COPD who require surgery for lung cancer.

## Supplementary Information

**Additional file 1: Table 1**.

**Additional file 2: Table 2**.

**Additional file 3: Table 3**.

**Additional file 4: Fig. 1**. Flow chart of patient selection.

## Data Availability

All data generated or analyzed during this study are included in this published article and its Additional information files.

## Abbreviations

COPD: Chronic obstructive pulmonary disease
LAMA: Long-acting muscarinic antagonist
LABA: Long-acting 2 agonist
ICS: Inhaled corticosteroid
AFL: Airflow limitation
FEV_1_: Forced expiratory volume in 1s
FVC: Forced vital capacity
GOLD: Global initiative for chronic obstructive lung disease
SD: Standard deviation
CI: Confidence interval
OS: Overall survival
BD: Bronchodilator
FEV_1_%pred: Percentage of predicted expiratory volume in 1s
DFS: Disease-free survival
