# Supplementary material for: Prognostic and functional impact of perioperative LAMA/LABA inhaled therapy in patients with lung cancer and chronic obstructive pulmonary disease
Source: BMC Pulm Med. 2021 May 21;21:174. doi: 10.1186/s12890-021-01537-z (PMC8139148; doi:10.1186/s12890-021-01537-z)
Supplement: Supplementary file 1 — Additional file 1: Table 1. [file 12890_2021_1537_MOESM1_ESM.docx]

Supplementary Table 1. Components of perioperative inhaled therapy and inhalation period

| **Type of inhaled bronchodilator** | **No (%)** |
| --- | --- |
| **LAMA/LABA** |  |
| Glycopyrronium bromide/indacaterol maleate | 50 (78.1) |
| Tiotropium bromide hydrate + formoterol fumarate hydrate | 7 (10.9) |
| Tiotropium bromide hydrate + indacaterol maleate | 4 (6.3) |
| Umeclidinium bromide/vilanterol trifenatate | 2 (3.1) |
| Tiotropium bromide hydrate/olodaterol hydrochloride | 1 (1.6) |
| **LAMA** |  |
| Tiotropium bromide hydrate | 23 (100) |

| **Inhalation period, days (range)** | | | | |
| --- | --- | --- | --- | --- |
|  | Preoperative | | Postoperative | |
| **LAMA/LABA** | 27.7 (14–91) | *p*=0.500 | 396.6 (41–1604) | *p*=0.002 |
| **LAMA** | 24.5 (14–45) |  | 827.2 (40–2820) |  |
| COPD: chronic obstructive pulmonary disease; LAMA: long-acting muscarinic antagonists; LABA: long-acting β2 –agonists; BD: bronchodilator | | | | |
