# Supplementary material for: Prognostic and functional impact of perioperative LAMA/LABA inhaled therapy in patients with lung cancer and chronic obstructive pulmonary disease
Source: BMC Pulm Med. 2021 May 21;21:174. doi: 10.1186/s12890-021-01537-z (PMC8139148; doi:10.1186/s12890-021-01537-z)
Supplement: Supplementary file 2 — Additional file 2: Table 2. [file 12890_2021_1537_MOESM2_ESM.docx]

Supplementary Table 2. Cause of death in patients who died during the study period

| **Cause of death** | **LAMA/LABA** | **LAMA** | **No-BD** |
| --- | --- | --- | --- |
|  | **n=5** | **n=15** | **n=20** |
| **Lung cancer** | 3 (60.0) | 10 (66.7) | 15 (75.0) |
| **Other** |  |  |  |
| Pneumonia | - | 2 (13.3) | 3 (15.0) |
| Heart failure | - | - | 1 (5.0) |
| Cerebral infarction | 1 (20.0) | - | - |
| Accidental death | 1 (20.0) | 3 (20.0) | - |
| Other cancer | - | - | 1 (5.0) |
| Data are presented as n (%)  COPD: chronic obstructive pulmonary disease; LAMA: long-acting muscarinic antagonists; LABA: long-acting β2 –agonists; BD: bronchodilator | | | |
