# Supplementary material for: Prognostic and functional impact of perioperative LAMA/LABA inhaled therapy in patients with lung cancer and chronic obstructive pulmonary disease
Source: BMC Pulm Med. 2021 May 21;21:174. doi: 10.1186/s12890-021-01537-z (PMC8139148; doi:10.1186/s12890-021-01537-z)
Supplement: Supplementary file 3 — Additional file 3: Table 3. [file 12890_2021_1537_MOESM3_ESM.docx]

Supplementary Table 3. Univariate and multivariate analysis of favorable factors for postoperative prognosis in all patients.

A. Analysis of favorable factors for overall survival

| **Clinicopathologic variable** | **Univariate analysis** | | | **Multivariate analysis** | | |
| --- | --- | --- | --- | --- | --- | --- |
|  | **RR** | **95%CI** | ***p* value** | **RR** | **95%CI** | ***p* value** |
| **Age (< 75 y)** | 0.80 | 0.40-1.69 | 0.544 | - | - | - |
| **Histology**: |  |  | 0.001 |  |  | 0.002 |
| Squamous cell carcinoma | 1.00 | reference | - | 1.00 | reference | - |
| Adenocarcinoma | 0.30 | 0.14-0.58 | <0.001 | 0.293 | 0.14-0.59 | <0.001 |
| Other | 0.24 | 0.04-0.87 | 0.036 | 0.264 | 0.04-1.02 | 0.053 |
| **Severely of AFL (mild)** | 0.85 | 0.44-1.61 | 0.614 | - | - | - |
| **Lymph node metastasis (absent)** | 0.48 | 0.25-0.99 | 0.049 | 0.308 | 0.95-9.73 | 0.059 |
| **Pathologic stage:** |  |  | 0.033 |  |  | 0.023 |
| I | 1.00 | reference | - | 1.00 | reference | - |
| II | 2.21 | 0.91-4.82 | 0.074 | 3.09 | 0.97-8.20 | 0.055 |
| III | 2.96 | 1.15-6.71 | 0.026 | 7.27 | 1.85-24.6 | 0.006 |
| **Bronchodilator** |  |  | 0.015 |  |  | 0.073 |
| LAMA/LABA | 1.00 | reference | - | 1.00 | reference | - |
| LAMA or No-BD | 2.89 | 1.20–8.57 | - | 2.27 | 0.93-6.79 | - |
| AFL: air flow limitation; LAMA: long-acting muscarinic antagonists; LABA: long-acting β2 –agonists; BD: bronchodilator; RR: relative risk; CI: confidence interval | | | | | | |

B. Analysis of favorable factors for disease-free survival

| **Clinicopathologic variable** | **Univariate analysis** | | | **Multivariate analysis** | | |
| --- | --- | --- | --- | --- | --- | --- |
|  | **RR** | **95%CI** | ***p* value** | **RR** | **95%CI** | ***p* value** |
| **Age (< 75 y)** | 0.71 | 0.34-1.35 | 0.309 | - | - | - |
| **Histology**: |  |  | 0.017 |  |  | 0.151 |
| Squamous cell carcinoma | 1.00 | reference | - | 1.00 | reference | - |
| Adenocarcinoma | 0.43 | 0.23-0.77 | 0.004 | 0.52 | 0.27-1.01 | 0.052 |
| Other | 0.51 | 0.14-1.37 | 0.198 | 0.67 | 0.18-1.91 | 0.475 |
| **Severely of AFL (mild)** | 0.948 | 0.54-1.67 | 0.854 | - | - | - |
| **Lymph node metastasis (absent)** | 0.342 | 0.19-0.63 | <0.001 | 0.40 | 0.13-1.32 | 0.115 |
| **Pathologic stage** |  |  | <0.001 |  |  | 0.005 |
| I | 1.00 | reference | - | 1.00 | reference | - |
| II | 2.47 | 1.17-4.88 | 0.019 | 3.08 | 1.12-7.25 | 0.031 |
| III | 4.86 | 2.32-9.65 | <0.001 | 9.56 | 2.56-32.4 | 0.001 |
| **Bronchodilator** |  |  | 0.007 |  |  | 0.018 |
| LAMA/LABA | 1.00 | reference | - | 1.00 | reference | - |
| LAMA or No-BD | 2.48 | 1.27-5.31 | - | 2.24 | 1.14-4.80 | - |
| AFL: air flow limitation; LAMA: long-acting muscarinic antagonists; LABA: long-acting β2 –agonists; BD: bronchodilator; RR: relative risk; CI: confidence interval | | | | | | |
