# Supplementary figures and images for: Prognostic and functional impact of perioperative LAMA/LABA inhaled therapy in patients with lung cancer and chronic obstructive pulmonary disease
Source: BMC Pulm Med. 2021 May 21;21:174. doi: 10.1186/s12890-021-01537-z (PMC8139148; doi:10.1186/s12890-021-01537-z)

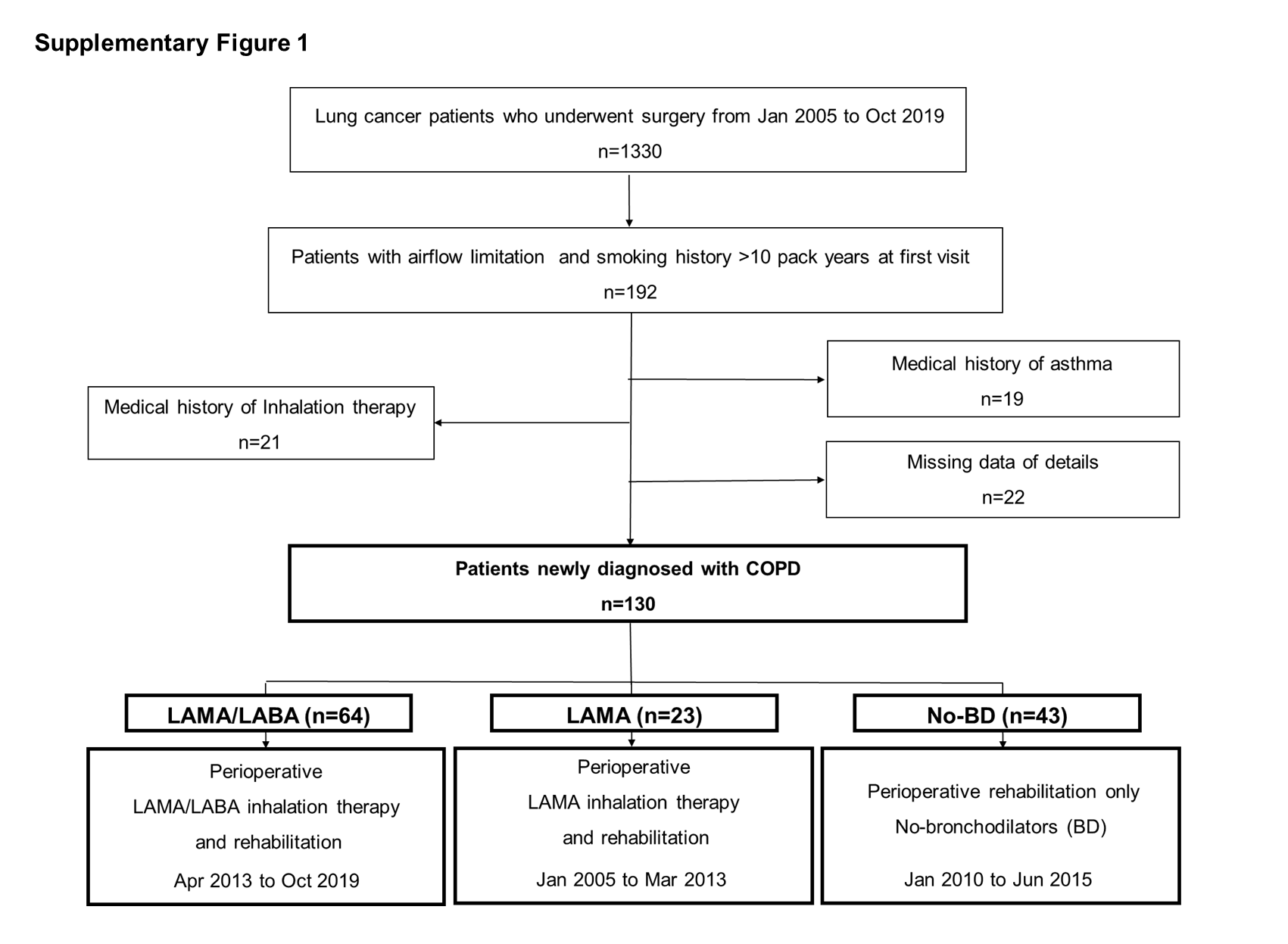

Supplement: Supplementary file 4 — Additional file 4: Fig. 1. Flow chart of patient selection. [file 12890_2021_1537_MOESM4_ESM.tif]
